# Foraging investment in a long‐lived herbivore and vulnerability to coursing and stalking predators

**DOI:** 10.1002/ece3.4489

**Published:** 2018-09-17

**Authors:** David Christianson, Matthew S. Becker, Angela Brennan, Scott Creel, Egil Dröge, Jassiel M'soka, Teddy Mukula, Paul Schuette, Daan Smit, Fred Watson

**Affiliations:** ^1^ School of Natural Resources and the Environment University of Arizona Tucson Arizona; ^2^ Zambian Carnivore Programme Mfuwe Eastern Province Zambia; ^3^ World Wildlife Fund Washington District of Columbia; ^4^ Department of Ecology Montana State University Bozeman Montana; ^5^ 5Wildlife Conservation Research Unit Department of Zoology, The Recanati‐Kaplan Centre University of Oxford The Recanati‐Kaplan Centre; ^6^ Department of National Parks and Wildlife Chilanga Lusaka Province Zambia; ^7^ Alaska Center for Conservation Science Anchorage Alaska; ^8^ School of Natural Sciences California State University‐Monterey Bay Seaside California

**Keywords:** courser, foraging, incisor, senescence, stalker, tooth wear

## Abstract

Allocating resources to growth and reproduction requires grazers to invest time in foraging, but foraging promotes dental senescence and constrains expression of proactive antipredator behaviors such as vigilance. We explored the relationship between carnivore prey selection and prey foraging effort using incisors collected from the kills of coursing and stalking carnivores. We predicted that prey investing less effort in foraging would be killed more frequently by coursers, predators that often exploit physical deficiencies. However, such prey could expect delayed dental senescence. We predicted that individuals investing more effort in foraging would be killed more frequently by stalkers, predators that often exploit behavioral vulnerabilities. Further these prey could expect earlier dental senescence. We tested these predictions by comparing variation in age‐corrected tooth wear, a proxy of cumulative foraging effort, in adult (3.4–11.9 years) wildebeest killed by coursing and stalking carnivores. Predator type was a strong predictor of age‐corrected tooth wear within each gender. We found greater foraging effort and earlier expected dental senescence, equivalent to 2.6 additional years of foraging, in female wildebeest killed by stalkers than in females killed by coursers. However, male wildebeest showed the opposite pattern with the equivalent of 2.4 years of additional tooth wear in males killed by coursers as compared to those killed by stalkers. Sex‐specific variation in the effects of foraging effort on vulnerability was unexpected and suggests that behavioral and physical aspects of vulnerability may not be subject to the same selective pressures across genders in multipredator landscapes.

## 1 INTRODUCTION

Vulnerability to predation has long been viewed as a strong driver of behavioral, morphological, physiological, and ontological adaptations in ungulate prey (Caro, 2005; Estes, 1991; Torriani, Vannoni, & McElligott, 2006). On ecological timescales, many characteristics of individual prey and local predators can affect probabilities in the predation sequence (encounter, attack|encounter, kill|attack) whose product determines an individual's risk of predation (Bateman, Vos, & Anholt, 2014; Macarthur & Pianka, 1966; Murray, Boutin, Odonoghue, & Nams, 1995; Scheel, 1993; Schmitz, 2008; Streams, 1994). Much of our current understanding of vulnerability has focused on the role of hunting methods near the terminal point of the predation sequence. For example, coursers (e.g., large canids and hyenids) will often search conspicuously for prey and select an individual for attack once flight ensues (Creel, 2001; Lingle & Pellis, 2002). Stalkers and ambush predators (e.g., felids) will often encounter unaware prey in terrain that provides concealing cover and select an oblivious target to chase or ambush (Fitzgibbon, 1989; Mills, Broomhall, & du Toit, 2004).

Hunting method also interacts with prey characteristics to influence vulnerability. For example, in the Greater Yellowstone Ecosystem, wolves (*Canis lupus*) were 7.1 times more likely to kill elk (*Cervus elaphus*) and mule deer (*Odocoileus hemionus*) in poor physical condition than mountain lions, while mountain lions (*Puma concolor*) killed prey occupying more complex terrain (Atwood, Gese, & Kunkel, 2007). In Serengeti, wild dogs (*Lycaon pictus)* killed Thomson's gazelles (*Eudorcas thomsonii*) in worse body condition than cheetahs (*Acinonyx jubatus*; Fitzgibbon & Fanshawe, 1989) while cheetahs in Kruger National Park were more successful killing prey using woodland/grassland ecotones that facilitated stalking and chasing prey (Mills et al., 2004). Vulnerability to coursers appears more strongly dependent on variation amongst prey individuals in the probability of a kill|attack when prey condition likely matters most. Vulnerability to stalkers appears more strongly influenced by variation in the probability of encounter where behavioral differences in habitat selection could exert strong effects (but see Fitzgibbon, 1989 for evidence that stalking cheetahs encountering a choice of two equidistant gazelles tended to hunt the less vigilant one suggesting variation in probability of attack|encounter driven by prey behavior may also be important to stalkers).

Despite unique hunting methods that appear to exploit different vulnerabilities in prey, juveniles and adults with physical deficiencies complicated by senescence are often overrepresented in the kills of both coursers and stalkers (Estes & Estes, 1979; Gervasi, Nilsen, & Linnell, 2015; McLellan et al., 2012; Metz, Smith, Vucetich, Stahler, & Peterson, 2012; Montgomery, Vucetich, Peterson, Roloff, & Millenbah, 2013; Winnie & Creel, 2007). This suggests prey condition may interact with several factors that could influence the probability of encounter, attack, or kill, raising questions about the ultimate and proximate drivers of vulnerability. Adding complexity, very young and very old individuals often represent only a small subset of the prey population, particularly outside the parturition period. Robust adults are also routinely killed by both predator types (Eberhardt, White, Garrott, & Houston, 2007; Vucetich, Smith, & Stahler, 2005). It is unclear how condition is related to factors that determine vulnerability and whether these mechanisms apply to prime‐age prey.

Prey carcasses represent the endpoint of predation and often carcass physical traits (e.g., sex, age, and condition) provide the only data to test hypotheses about vulnerability preceding death. Consequently, many drivers of predation, in robust individuals or otherwise, remain little explored. For example, foraging effort likely influences the probabilities of encounter, attack|encounter, and kill|attack, but this process is poorly understood in ungulates. Large herbivores must invest 30%–50% of their day foraging and accessing water (Owen‐Smith, 1992; Ruckstuhl, 1998) often at the expense of antipredator behaviors such as vigilance (Creel et al., 2017) or spatiotemporal avoidance of predators (Valeix et al., 2009). Foraging effort varies amongst prime‐age individuals and correlates positively with condition and body size (Gélin, Coulson, & Festa‐Bianchet, 2016; Hamel & Côté, 2009), but drivers of individual variation in foraging effort are poorly understood. Herbivores investing strongly in foraging effort and in relatively good condition might rarely express antipredator behaviors that compete with foraging. If condition plays an important role in determining vulnerability in all classes of prey, we might predict that prime‐age prey killed by coursers forage less than prime‐age prey killed by stalkers. We might also predict this pattern if foraging effort trades off with proactive antipredator behaviors such as vigilance that may be most effective for deterring attacks by stalkers (Bednekoff & Ritter, 1994; Boving & Post, 1997; Creel, Schuette, & Christianson, 2014; Fitzgibbon, 1989; Liley & Creel, 2008; Winnie & Creel, 2007). In systems where prime‐age prey are killed by both predator types, examining whether individuals killed by coursers foraged less than individuals killed by stalkers could be insightful for developing a more general theory of prey vulnerability in ungulates.

Descriptions of individual foraging effort in any ungulate are sparse due to the difficulty in collecting such data. Carranza, Alarcos, Sanchez‐Prieto, Valencia, and Mateos (2004) first proposed that herbivore tooth wear could be interpreted as a measure of long‐term foraging investment because erosion of dental tissue is an irreparable consequence of clipping and chewing plants. They showed that male red deer (*Cervus elaphus*) had shorter life spans but more rapid growth rates and larger body size than females. However, dental sexual dimorphism was weak, and male molars wore at a 74% faster rates processing the greater intake of forage needed to support rapid growth and large body size. Using morphological data from two populations of red deer, Pérez‐Barbería et al. (2015) further showed that within sexes, molar wear correlated positively with mandible length (an index of body size) but also negatively with longevity. In the highly sexually dimorphic Svalbard reindeer (*Rangifer tarrandus platyrhynchus*), Veiberg et al. (2007) found that even females experienced a grow fast, wear fast trade‐off. Importantly, they also showed that larger females wore teeth at faster rates yet maintained rumen particle size important for efficient digestion. They suggested that larger individuals not only foraged more but likely ruminated more as they aged to compensate for declining mastication efficiency. Here, we used age‐corrected wear of the permanent first incisor from adult, nonsenescent wildebeest as a measure of cumulative foraging effort preceding death by stalking (lion, cheetah) and coursing (spotted hyena, African wild dog) large carnivores, respectively.

## 2 MATERIALS AND METHODS

### 2.1 Study site

We conducted this study from 2010 to 2014 in Liuwa Plain National Park, a 3,242 km^2^ protected area in western Zambia composed almost entirely of the Western Zambezian Grasslands ecoregion (http://www.worldwildlife.org/ecoregions/at0724, accessed: 6‐16‐2016). Soils are sandy, poorly drained, and nutrient poor. Tree growth is largely limited by water saturation of soils during the west season (November‐April) and extensive wildfires during the dry season (May–October), but a few small, isolated stands of trees and shrub occur throughout the park. The park is bounded to the east, south, and north by the floodplain of the Zambezi River and associated tributaries.

The herbivore community is dominated by a migratory population of wildebeest (*Connechaetes taurinu*s) estimated by aerial survey in 2015 at 35,000 animals (APN 2015) with local densities as high as 60.8 individuals/km^2^ (M'soka, Creel, Becker, & Murdoch, 2017). Migratory zebra (*Equus quagga*) are the next most abundant prey species (<8.1 individuals/km^2^) followed by oribi (*Ourebia ourebi*), tsessebe (*Damaliscus lunatus*), and lechwe (*Kobus leche*). Spotted hyena (*Crocuta crocuta*) are overwhelmingly the most abundant large carnivore persisting in >5 major clans of ~233 hyenas during this study (M'soka, Creel, Becker, & Droge, 2016). Only two wild dogs (*Lycaon pictus*) packs were known to persist, ranging from 7 to 22 dogs, and 17 cheetah *(Acinonyx jubatus*) were individually identified during the course of the study. Lions (*Panthera leo*) were reduced to a single lioness by 2003 and additional reintroductions increased the population to three to five lions during the study period. Although stalkers were relatively rare compared to coursers, both lions and cheetah could be regularly followed using radio collars to locate their kills (Droge, Creel, Becker, & M'soka, 2017).

Wildebeest were the most important prey for three of the four carnivores, comprising 92% of hyena kills, 90% of lion kills, 59% of wild dog kills, and 30% of cheetah kills (Creel et al., 2017). Cheetahs and wild dogs preyed heavily on wildebeest calves (83% and 75% of wildebeest killed, respectively). Lions and hyenas preyed heavily on adults (86% and 89% of wildebeest killed, respectively) and mainly killed males—half of all prey killed by lion and hyena were adult male wildebeest (50% and 49%, respectively).

### 2.2 Carcass detection and incisor collection

**Figure 1:**
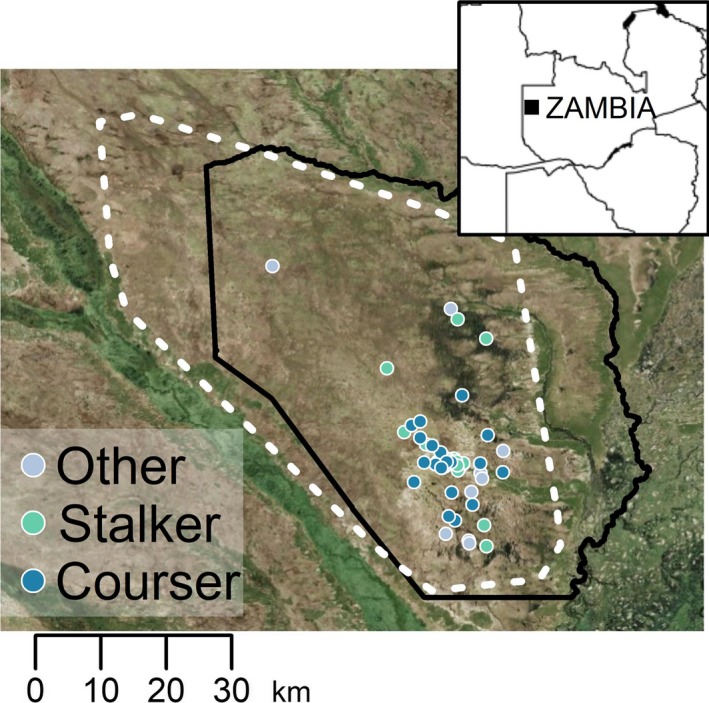
Spatial distribution of 59 carcasses contributing incisors from wildebeest killed by stalkers (cheetah and lions), coursers (spotted hyena and wild dogs), and other causes (primarily unknown, poaching, and fences) in Liuwa Plain National Park (solid line), western Zambia. The annual extent of the migratory wildebeest population is shown as a minimum convex hull of GPS collar points from six adult female wildebeest (broken line) moving from dry season (May–October) range in the southeast to wet season (November–April) range in the northwest in a clockwise manner. Seven wildebeest (three courser‐killed and four others) with missing location data are not shown

**Figure 2:**
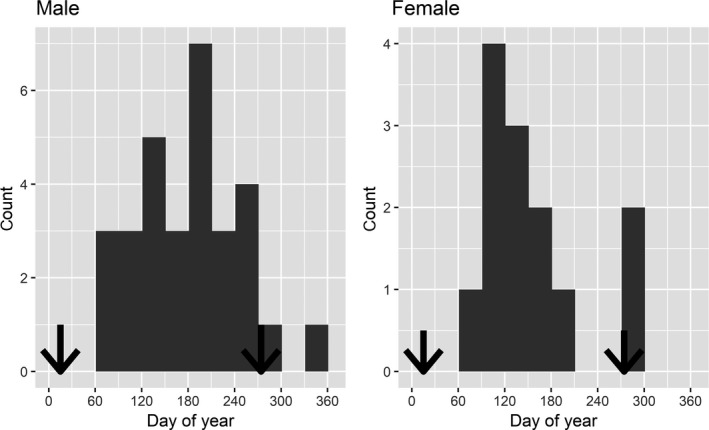
Timing of wildebeest mortalities used in the analysis of tooth wear. The estimated peak in rutting in males (15 January) and parturition in females (1 October) are shown by the left and right arrows, respectively

We collected incisors from adult (permanent I1 erupted) wildebeest carcasses encountered opportunistically or while following hunting lions, wild dogs, cheetah, and spotted hyena. While we attempted to identify the specific carnivore species responsible for a kill, for the purposes of this study, we were primarily interested in whether wildebeest were killed by a courser or stalker. Using broader predator‐type categories should have reduced misclassifications and Type II errors (which was not an issue as we detected significant effects of predator type, see Results). We excluded carcasses that appeared to have been dead for more than 24 hours. Mean time lag between estimated death and carcass sampling was 5.9 hr (±1.8 95% CI). Upon detecting a carcass, we scanned the area visually with binoculars followed by a radio‐scan for all VHF‐collared carnivores. If any carnivores were present at the carcass, we waited until feeding finished before approaching the carcass indirectly in a tightening spiral to scan the ground for carnivore tracks and wildebeest tracks indicating a chase or struggle (indicative of predation). Predation was distinguished from scavenging primarily by direct observation of the kill or based on evidence of flowing blood at the time of death. In less ambiguous cases, we detected predator tracks, signs of a chase, hair clumps, and blood trails. We also examined the skin of wildebeest carcasses to identify bite marks on nose and throat that commonly occurs when lions and cheetah asphyxiate their prey. In 48% of carcasses (a), the kill was directly observed while following a hunting carnivore, (b) the kill was audibly detected moments before visual confirmation, or (c) the carnivore was directly observed on the carcass along with supporting evidence for predation as the cause of death. Due to the large geographic extent of this migratory wildebeest population and because of seasonal flooding, our sampling was spatially (Figure 1) and temporally (Figure 2) restricted.

**Figure 3:**
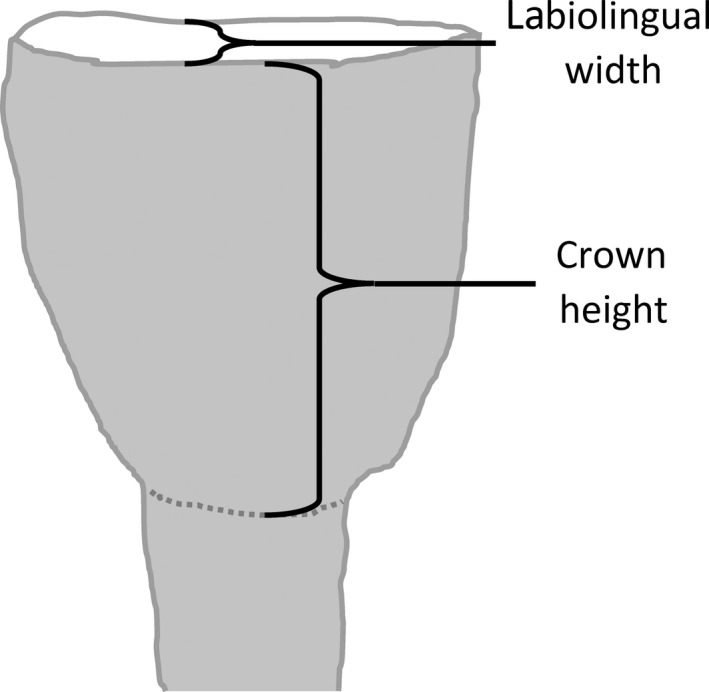
Measurement of the first permanent incisor of wildebeest to define tooth wear. When the first incisor erupts, the labioincisal and linguoincisal edges are joined at the apex of the incisor, but these edges migrate apart as the incisor wears into a broader occlusal platform. Labiolingual width is measured between these edges, where the mesiolingual groove terminates on the linguoincisal edge. Crown height is measured on the labial side, from the labioincisal edge to the termination of the crown enamel at the top of the root

At 59 carcasses, we located the mandible and extracted both first incisors. Both first incisors could not always be collected owing to missing or partially consumed mandibles. Extracted incisors were placed in a coin envelope and stored for up to 12 months. A single individual measured the labiolingual width and incisor height of the occlusal surface of the incisor using a digital Vernier caliper (Figure 3). Cementum annuli of each incisor were counted to estimate age at Matson's Laboratory (Manhattan, Montana, USA) where the condition of the teeth for age estimation was considered excellent. Because this region experiences a single distinct wet season in each year, we assumed each annulus indicated 1 year of growth since incisor eruption. We increased precision of the age estimates to 0.1 years according to the date of death and an estimated birth date of October 1, the approximate birth peak for wildebeest in the Greater Liuwa Ecosystem (M'soka et al., 2017).

### 2.3 Data analysis

We collected measurements from both the left and right first incisor when available and used the mean for analysis. Paired t tests showed no difference between left and right incisors (mean difference labiolingual width: −0.04 mm ± 0.16 95% CI, *n* = 22, *p* = 0.603 and mean difference crown height: 0.05 mm ± 0.37 95% CI, *n* = 22, *p* = 0.761). Crown height decreases and labiolingual width increases as an incisor wears (Attwell, 1980; Christianson, Gogan, Podruzny, & Olexa, 2005; Spinage, 1973) and tests using both measurements would be largely redundant. Labiolingual width showed more unexplained variation after accounting for age (see Section 2.1). Further, we failed to detect sexual dimorphism in labiolingual width (see below), but crown height has been shown to be more sensitive to sexual dimorphism than labiolingual width in large grazers (Christianson et al., 2005). For these reasons, we focused on labiolingual width in our analysis; however, we also confirmed that using crown height in our modeling had no effect on the relative magnitude or direction of any effect size. We regressed labiolingual width on age, cause of death, gender, and their interaction. We used *F*‐ratio tests and adjusted *r* ^2^ to identify significant effects in models.

Increasing wear with age was expected and was not of primary interest, but the precise relationship between wear and age was not known in advance. We first visually inspected data using scatterplots to identify outliers and explore the potential for nonlinear relationships between wear and age. We noted one extreme outlier for incisor labiolingual width and crown height in a 7‐year‐old animal that strongly suggested the incisor crown fractured early in life. After removing this animal, we tested for nonlinear relationships by fitting regressions of labiolingual width onto age and age with its square. We found no significant improvement in fit over the linear form (increase in *r* ^2^ with quadratic form: 0.002, *F*_1, 56_ = 1.23, *p* = 0.273).

Wildebeest males and females differ significantly in mass (Estes, 1991) and probably longevity (Loison, Festa‐Bianchet, Gaillard, Jorgenson, & Jullien, 1999), yet tooth size scales allometrically (kg^0.25–0.40^) and tooth size differences between highly dimorphic sexes can be small (Spaeth et al., 2001) or essentially nil (Carranza et al., 2004). We assumed direct comparisons between male and female incisor measurements were possible after testing for dimorphism in several ways. We found no support for the addition of an additive gender effect to a linear model of labiolingual width regressed onto age using data from all wildebeest (*F*_1,52_ = 0.002, *p* = 0.965). Likewise, we found no evidence for a gender effect interacting with age, that is, sex‐specific wear rates were not supported (*F*_2,51_ = 0.042, *p* = 0.958). We also measured the incisor root diameter at the base of the crown (which is not exposed to wear except in very old individuals) along both the sagittal and frontal planes and tested for a gender effect on root diameter (in addition to a linear age effect). We found no gender effect on incisor root diameter measured in either plane (*F*_1,18_ = 0.712, *p* = 0.41 and *F*_1,18_ = 1.164, *p* = 0.295), and the estimated effect size was in the opposite direction predicted if sexual dimorphism contributed significantly to our results—males showed an insignificant tendency for smaller incisor roots than females.

Very old individuals with highly advanced tooth wear can experience nutritional deficits due to reduced chewing efficiency (Pérez‐Barbería, Carranza, & Sánchez‐Prieto, 2015; von Hardenberg, Shipley, & Festa‐Bianchet, 2003). Nutritional deficits in these age classes could interact with vulnerability such that wildebeest killed by coursers would tend to show high levels of tooth wear, an effect that could mask any relationship between foraging effort and vulnerability. We concluded this was not a significant issue in our dataset for several reasons: (a) The oldest killed wildebeest in our sample was 11.9 years, far from the maximum age reported in other populations, for example, 18 years in South Africa (Attwell, 1980) and 18 years in Tanzania (Talbot & Talbot, 1963), (b) age distributions of wildebeest killed by coursers and stalkers were very similar (see Section 2.1), (c) all wildebeest in our sample possessed substantial incisor crowns at the time of death (see Section 2.1) and were likely several years from dental senescence (d) excluding all wildebeest in the oldest age class, ≥11.0 years (*n* = 5) or with <10 mm incisor crown height (*n* = 3) had no effect on our results.

## 3 RESULTS

### 3.1 Composition of the wildebeest incisor sample

**Figure 4:**
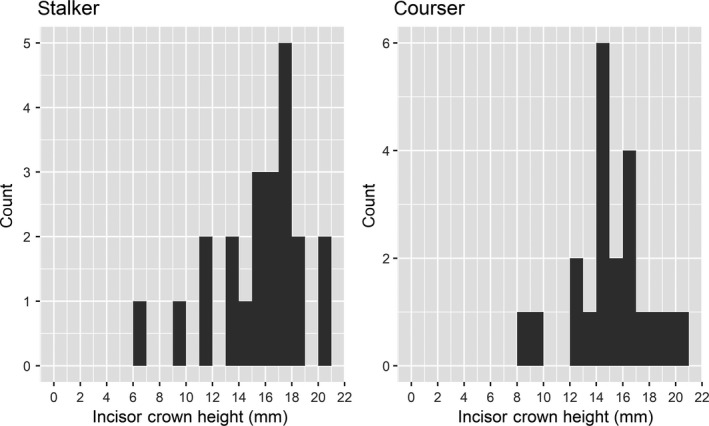
Composition of incisor crown heights from wildebeest killed by coursing and stalking carnivores in Liuwa Plain National Park, Zambia. Incisor crown heights near or less than zero indicate advanced dental senescence likely to negatively influence nutrient assimilation

We estimated cementum annuli age, labiolingual width, and crown height from 59 adult wildebeest incisors collected at carcasses. Of these 59 cases, we identified wildebeest gender at 55 carcasses, cause of death at 44 carcasses, and both gender and cause of death at 43 carcasses (five females and 16 males killed by coursers; eight females and 14 males killed by stalkers). Our sample included fewer incisors from female wildebeest than males (proportion female = 0.309, exact binomial test: *n* = 55, *p* = 0.006, Figure 2). There was no difference in the proportion of male:female incisors from stalker and courser mortalities (Pearson's chi‐square = 0.32, *p* = 0.573, *n* = 43). Wildebeest killed by stalkers spanned 3.5–11.9 years of age and wildebeest killed by coursers spanned 4.7–11.6 years with no difference in age between wildebeest killed by coursers and those killed by stalkers (mean difference = −0.20 ± 1.37 years, 95% CI, *t* = −0.30, *n* = 43, *p* = 0.768). Coursers killed female wildebeest 1.97 yrs older than males, but there was considerable uncertainty in this difference (± 3.46 yrs 95% CI, Welch's *t* = 1.50, *df* = 4.53, *p* = 0.198). Female and male wildebeest killed by stalkers were more similar in age (0.46 years ± 1.66 95% CI, Welch's *t* = −0.38, *df* = 13.27, *p* = 0.707. No wildebeest showed incisor wear progressing into the base of the crown or root that would have indicated advanced dental senescence affecting nutrient assimilation (Figure 4).

### 3.2 Effects of age, gender, and predator on incisor wear

Single factor linear regression using all wildebeest showed that substantial variation in labiolingual width was not explained by age (*r* ^2^_a_ = 0.405, *n* = 59). Restricting our analysis to wildebeest of known gender and cause of mortality (*n* = 43), we found support for a model with age, gender, and predator type over a model with only age (*F*_3,38_ = 4.14, *p* = 0.012). In this model, substantially more variation was explained (*r* ^2^_a_ = 0.542). Model coefficients showed labiolingual width increased by 0.31 mm per year (±0.09 95% CI). The main effect of gender and predator was comparable in magnitude, but the interaction term was nearly twice as large and in the opposite direction as the main effects (Table 1). Consequently, we tested predictions by estimating the mean response in wear to the effects of gender and predator type with age fixed at its mean (7.3 years). Females killed by stalkers had 0.80 mm more wear than females killed by coursers (5.09 mm ± 0.21 SE vs. 4.28 ± 0.28), while males killed by stalkers had 0.62 mm less wear than males killed by stalkers (4.49 ± 0.16 vs. 5.10 ± 0.15, Figure 6). While our sample size was small and the negative interaction between gender and predator type was unexpected, our power was high due to the magnitude of the interaction (Table 1, *α* = 0.002, 1 − β = 0.775).

**Table 1:** Coefficient estimates from a linear model of the incisor labiolingual width in male and female wildebeest killed by coursers (hyena and wild dogs) and stalkers (lions and cheetah) in Liuwa Plain National Park, Zambia

Model coefficient	Estimate	SE	*t*	*p*
Intercept	2.040	0.464	4.40	<0.001
Age (years)	0.306	0.043	7.06	<0.001
Sex (male)	0.820	0.321	2.56	0.014
Predator (stalker)	0.802	0.350	2.29	0.028
Sex:predator	−1.420	0.422	−3.36	0.002

## 4 DISCUSSION

**Figure 5:**
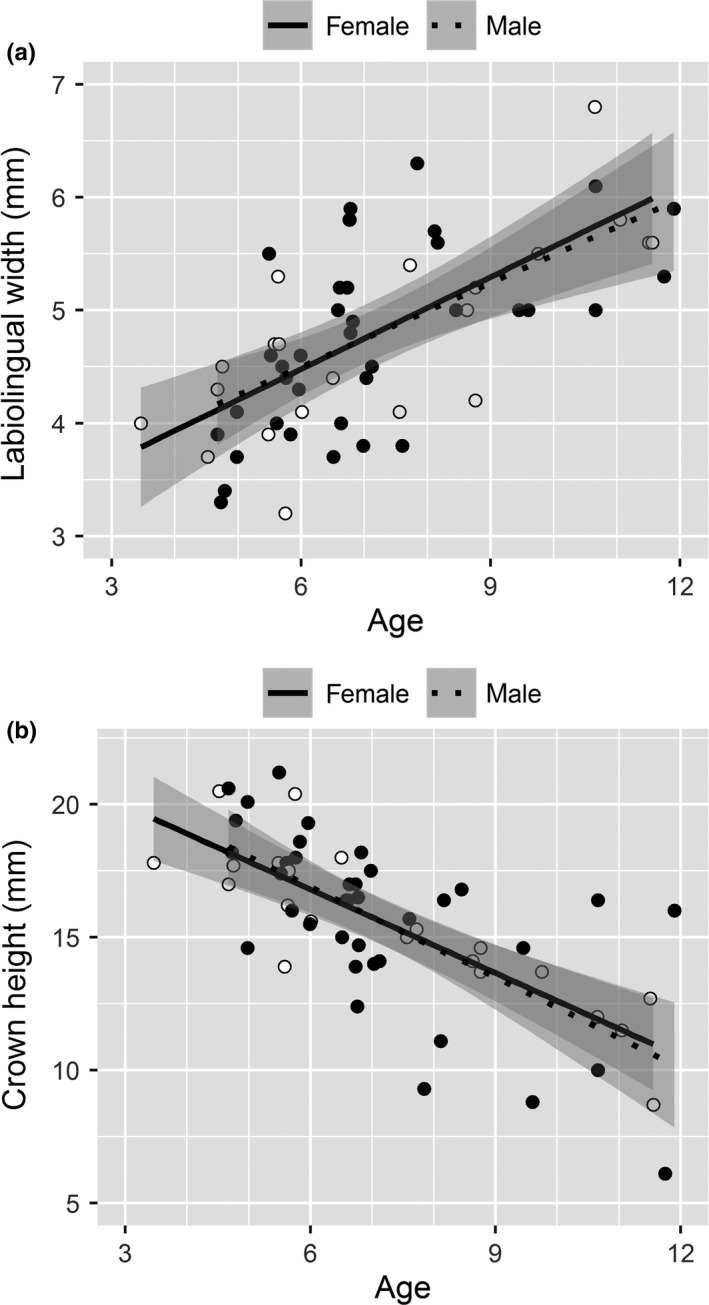
Progression of first incisor (a) labiolingual width and (b) crown height in (white points) female and (black points) male adult wildebeest in Liuwa Plain National Park, Zambia Fitted values (line) and 95% confidence limits (shaded area) from a linear model of wear on cementum annuli age for each gender are shown

**Figure 6:**
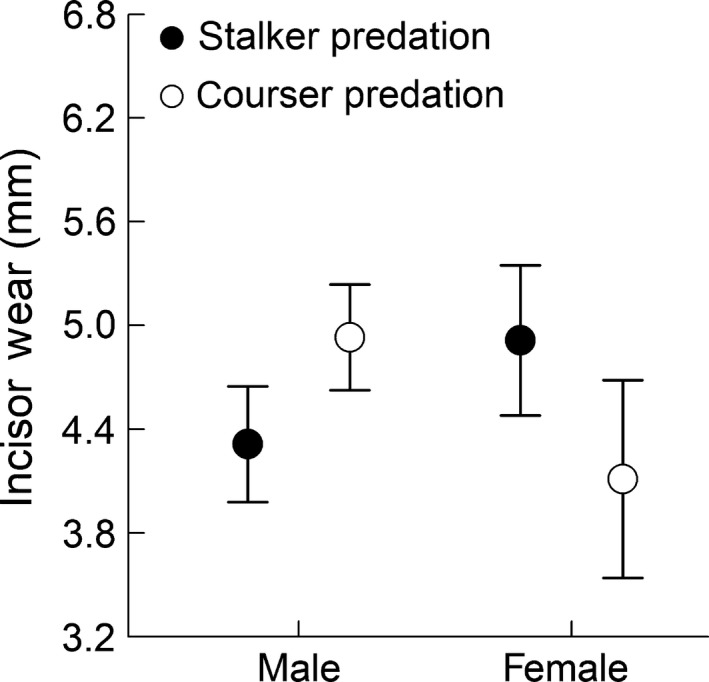
Expected first incisor labiolingual width (±95% CI) in wildebeest killed by large carnivores in Liuwa Plain National Park, Zambia. Expected width is estimated from linear model of width as a function of age, sex, and carnivore mortality type, with age fixed at its mean of 7.3 years. The vertical axis for wear spans the range of wear (3.2–6.8 mm) in wildebeest aged 3.5–11.9 years in this sample

Labiolingual width was highly variable within age classes of wildebeest killed by carnivores. Within each wildebeest gender, incisor wear suggested differences in foraging investment between stalker and courser mortalities equivalent to 2.4–2.6 years of foraging effort (Figures 5 and 6) or, alternately, the advancement of dental senescence by 2.4–2.6 years. Differences measured in years are likely biologically significant given that these animals averaged 7.3 years at death, females can breed most years in their adult life, and the maximum observed life span is only 11.9 years in this system and 18 years elsewhere (Attwell, 1980; Talbot & Talbot, 1963). Because incisors are used for clipping vegetation and not mastication, these differences likely arise from variation in forage cropping rates at sites selected for foraging that not only drive energy intake rates but also trade‐off with several antipredator tactics in large herbivores (Brivio, Grignolio, Brambilla, & Apollonio, 2014; Ferretti et al., 2014; Ruckstuhl, Festa‐Bianchet, & Jorgenson, 2003; Wilmshurst, Fryxell, & Colucci, 1999).

Differences in tooth wear amongst prey types could reflect spatial variation in availability of abrasive forages or abrasive forage contaminants (Hummel et al., 2011), alongside covariation with predation risk from coursing and stalking carnivores. However, the distribution of wildebeest kills by coursers and stalkers overlapped spatially (Figure 1) and was temporally restricted seasonally to the period between breeding and parturition (Figure 2). In other systems where variation in tooth wear has been found to correspond with spatial variation in habitat quality or availability of abrasive forages, differences only became apparent when compared across populations (Christianson et al., 2005; Garrott, Eberhardt, Otton, White, & Chaffee, 2002; Kojola, Helle, Huhta, & Niva, 1998; Skogland, 1988). Our spatially and temporally restricted sampling within this highly mobile population suggests fine‐scale variation in forage abrasiveness is unlikely to explain the patterns in tooth wear here. Other factors, such as individual variation in tooth hardness might also be involved, but these factors must also covary with predator type and gender to explain our results. Even in the presence other explanatory factors, variation in tooth wear arising from variable foraging effort, as has been seen in other herbivore populations (Pérez‐Barbería et al., 2015; Veiberg et al., 2007), suggests important trade‐offs with nutrient assimilation, vulnerability to predation, and longevity.

Consistent with our prediction, female wildebeest killed by stalkers had higher age‐corrected wear rates than females killed by coursers. Owing to the timing of deaths (Figure 2), few of the females in our sample were unlikely to be responding to any increased risk or lactation demands posed by the presence of neonatal calves. We cannot explain the exact mechanism whereby greater investment in foraging effort by females increased their vulnerability to stalkers relative to coursers. We suspect that greater foraging effort in this grazer increased the probabilities of encounter and attack|encounter by stalkers due to increased movement (Frair et al., 2005) or because Liuwa wildebeest have been shown to decrease grazing and increase vigilance in response to predator proximity (Creel et al., 2017). One or both of these mechanisms operating simultaneously could explain our results.

We predicted male investment in territorial defense (Estes, 1966; Sinclair & Arcese, 1995) could result in smaller differences in foraging effort between predator mortality types. We did not predict the observed, contrasting responses to predator types between genders (Table 1, Figure 6). Wildebeest often segregate by gender, and several theories have been advanced to explain sexual segregation in ungulates (Ruckstuhl, 2007). It may be that this segregation, or the factors driving segregation, explain the contrasting relationship with age‐corrected tooth wear and courser/stalker predation between the genders. Male wildebeest killed by coursers foraged the equivalent of 2.4 years more, over a mean 7.3‐year life span, than males killed by stalkers. Male ungulates can grow faster, peak, and decline in body size and reproductive output sooner, and die younger than females (Carranza et al., 2004) so foraging and antipredator tactics that delay dental senescence in males may be disfavored by selection. Adult male wildebeest are generally larger than females, and it may be that any relationship between vulnerability and body size is different for stalkers and coursers (Owen‐Smith & Mills, 2008). Male wildebeest also segregate into “bachelor” groups and year‐round solitary, territorial individuals (Estes, 1969). Territorial defense can restrict movement in males and place time constraints on both antipredator and foraging behaviors (Bro‐Jørgensen, Brown, & Pettorelli, 2008; Vrahimis & Kok, 1993). Males most heavily invested in territorial defense might be expected to show less tooth wear within an age class and also be more vulnerable to stalkers. Younger bachelors and territorial bulls might be expected to show differences in vulnerability to predator type due to differences in movement rates or investment in activities such as territorial defense (Vrahimis & Kok, 1993). However, male wildebeest killed by coursers showed a statistically insignificant tendency to be younger than wildebeest killed by stalkers in our sample (6.7 vs. 7.6 years, *df* = 18.90, Welch's *t* = 1.223, *p* = 0.236). Furthermore, tooth wear in male wildebeest killed by coursers was identical to females killed by stalkers and below levels indicative of dental senescence when nutrient assimilation is most likely to be affected (Peréz‐Barbería and Gordon 1998). Consequently, our results do not appear to be an artifact arising from (a) coursers exploiting older males than stalkers (b) coursers exploiting males in worse condition due to dental senescence, or (c) stalkers exploiting younger, more mobile bachelor bulls that may be prone to ambush.

Our inference is limited due to our inability to compare the tooth wear of any age‐sex class with that of nonpredated wildebeest. Testing these ideas further will require coupling morphological, behavioral, and physiological data, but as Carranza et al. (2004) noted, general investigations into tooth wear as a measure of variation in foraging investment between and within the sexes could provide valuable insight into life‐history trade‐offs. Our results suggest that any solution to the trade‐off between longevity and resource allocation to growth and reproduction from foraging should also consider the risk of predation from each predator type.

## CONFLICT OF INTEREST

None declared.

## AUTHOR CONTRIBUTIONS

DC conceived of the concept and conducted the data analysis and leading the writing. MB, DC, SC, and PS contributed to sampling design. All authors contributed to collecting data and revising the manuscript.

## DATA ACCESSIBILITY

Wildebeest incisor data are available on Dryad: TBD.
